# Added value of cardiovascular calcifications for prediction of recurrent cardiovascular events and cardiovascular interventions in patients with established cardiovascular disease

**DOI:** 10.1007/s10554-021-02164-9

**Published:** 2021-02-12

**Authors:** Cilie C. van ’t Klooster, Yolanda van der Graaf, Hendrik M. Nathoe, Michiel L. Bots, Gert J. de Borst, Frank L. J. Visseren, Tim Leiner, the UCC-SMART-study group, F. W. Asselbergs, H. M. Nathoe, G. J. de Borst, M. L. Bots, M. I. Geerlings, M. H. Emmelot, P. A. de Jong, T. Leiner, A. T. Lely, N. P. van der Kaaij, L. J. Kappelle, Y. M. Ruigrok, M. C. Verhaar, F. L. J. Visseren, J. Westerink

**Affiliations:** 1 Department of Vascular Medicine, University Medical Center Utrecht (UMCU), Utrecht University, PO Box 85500, 3508 GA Utrecht, The Netherlands; 2 Julius Center for Health Sciences and Primary Care, University Medical Center Utrecht (UMCU), Utrecht University, Utrecht, The Netherlands; 3 Department of Cardiology, University Medical Center Utrecht (UMCU), Utrecht University, Utrecht, The Netherlands; 4 Department of Vascular Surgery, University Medical Center Utrecht (UMCU), Utrecht University, Utrecht, The Netherlands; 5 Department of Radiology, University Medical Center Utrecht (UMCU), Utrecht University, Utrecht, The Netherlands

**Keywords:** Stable cardiovascular disease, Recurrent cardiovascular events and interventions, Added prognostic value, Cardiovascular calcification scores

## Abstract

The purpose is to investigate the added prognostic value of coronary artery calcium (CAC), thoracic aortic calcium (TAC), and heart valve calcium scores for prediction of a combined endpoint of recurrent major cardiovascular events and cardiovascular interventions (MACE +) in patients with established cardiovascular disease (CVD). In total, 567 patients with established CVD enrolled in a substudy of the UCC-SMART cohort, entailing cardiovascular CT imaging and calcium scoring, were studied. Five Cox proportional hazards models for prediction of 4-year risk of MACE + were developed; traditional CVD risk predictors only (model I), with addition of CAC (model II), TAC (model III), heart valve calcium (model IV), and all calcium scores (model V). Bootstrapping was performed to account for optimism. During a median follow-up of 3.43 years (IQR 2.28–4.74) 77 events occurred (MACE+). Calibration of predicted versus observed 4-year risk for model I without calcium scores was good, and the c-statistic was 0.65 (95%CI 0.59–0.72). Calibration for models II–V was similar to model I, and c-statistics were 0.67, 0.65, 0.65, and 0.68 for model II, III, IV, and V, respectively. NRIs showed improvement in risk classification by model II (NRI 15.24% (95%CI 0.59–29.39)) and model V (NRI 20.00% (95%CI 5.59–34.92)), but no improvement for models III and IV. In patients with established CVD, addition of the CAC score improved performance of a risk prediction model with classical risk factors for the prediction of the combined endpoint MACE+ . Addition of the TAC or heart valve score did not improve risk predictions.

**Supplementary Information:**

The online version contains supplementary material available at (10.1007/s10554-021-02164-9).

## Introduction

Cardiovascular calcification scores are related to the risk of incident cardiovascular disease, independent of traditional cardiovascular risk factors [1–3]. Whether calcification is merely a consequence of the atherosclerotic process, or that it is causally related to cardiovascular disease (CVD), is debated [4, 5]. Intimal calcium deposition is even hypothesized to act as a plaque stabilizer, preventing acute atherosclerotic events [5]. Regardless of which hypothesis may hold its premise, calcium scores can be regarded as a marker of total plaque burden, [6] and could thereby reflect an individual’s risk of developing cardiovascular disease.

In patients without established cardiovascular disease, addition of coronary artery calcium scores to risk prediction models provides more accurate risk predictions, [7–9] with improvements in c-statistics ranging from 0.05 to 0.13 and reported net reclassification index (NRI) ranging from 14 to 25% [7]. Furthermore, current guidelines for primary prevention recommend to consider CAC scoring in patients with predicted 10-year risk of fatal cardiovascular disease around 5% or 10% thresholds, in order to reclassify patients and thereby aid in decision making regarding preventive treatment [10]. In addition to coronary artery calcium scores and traditional risk factors, thoracic aorta calcification scores did not improve risk prediction of all-cause mortality and cardiovascular events during a mean follow-up of 8.0 (± 1.5) years, [11] and neither did extra-coronary artery calcium scores, including thoracic aortic calcification, aortic valve and mitral annulus calcification for the prediction of stroke during a median follow-up of 12.1 years [12] in patients without cardiovascular disease.

In secondary prevention, patients with established cardiovascular disease are, on average, classified as high to very high risk patients [10]. However, distribution of predicted 10-year risk of recurrent cardiovascular disease risk varies widely in these patients [13]. Risk prediction models to estimate the risk of recurrent cardiovascular disease in these patients are available, [14, 15] and can provide a basis for intensifying treatment, or conversely refraining from intensifying preventive treatment, and give accurate prognostic information for patients. Furthermore, particularly in patients with established cardiovascular disease, calcium scores are often available as CT-imaging of the chest is often performed in these patients for various diagnostic indications.

The aim of the current study is to investigate the potential added predictive value of coronary artery, thoracic aorta, and heart valve calcification scores, on top of classical risk factors, for the prediction of a combined endpoint of recurrent major cardiovascular events (MACE) and cardiovascular interventions in patients with established cardiovascular disease.

## Methods

### Study population

Patients originated from a subcohort of the Utrecht Cardiovascular Cohort-Second Manifestation of ARTerial disease (UCC-SMART) cohort. The UCC-SMART cohort is an ongoing prospective cohort study starting from 1996, including 18 to 79-year-old patients referred to the University Medical Center Utrecht (UMCU), the Netherlands, with vascular disease or marked risk factors. Focus of the study is to gain insight in occurrence and risk factors of (recurrent) arterial disease in a high-risk population. Study design and rationale have been described in detail previously [16]. From August 2012, patients enrolled in the UCC-SMART cohort were invited to participate in the subcohort, consisting of cardiac non-contrast enhanced computed tomography (CT) and computed tomography angiography (CTA) of the heart, and the carotids to the circle of Willis. Exclusion criteria were known allergy to iodine containing contrast, reduced renal function (estimated glomerular filtration rate (eGFR) < 60 ml/min/1.73 m^2^), previous exposure to CT radiation for scientific purposes, or any other contra-indications for contrast enhanced CT. The institutional board of the UMCU approved the study and all participants provided written informed consent. For the current study, 567 patients with established cardiovascular disease at baseline enrolled in the UCC-SMART substudy with CT imaging were included. Definitions of established cardiovascular disease (coronary heart disease, cerebrovascular disease, or peripheral artery disease), predictors, and the endpoint recurrent cardiovascular events and cardiovascular interventions are described in detail in Supplemental Table S1.

### CT-scan protocol and image analysis

**Fig. 1:**
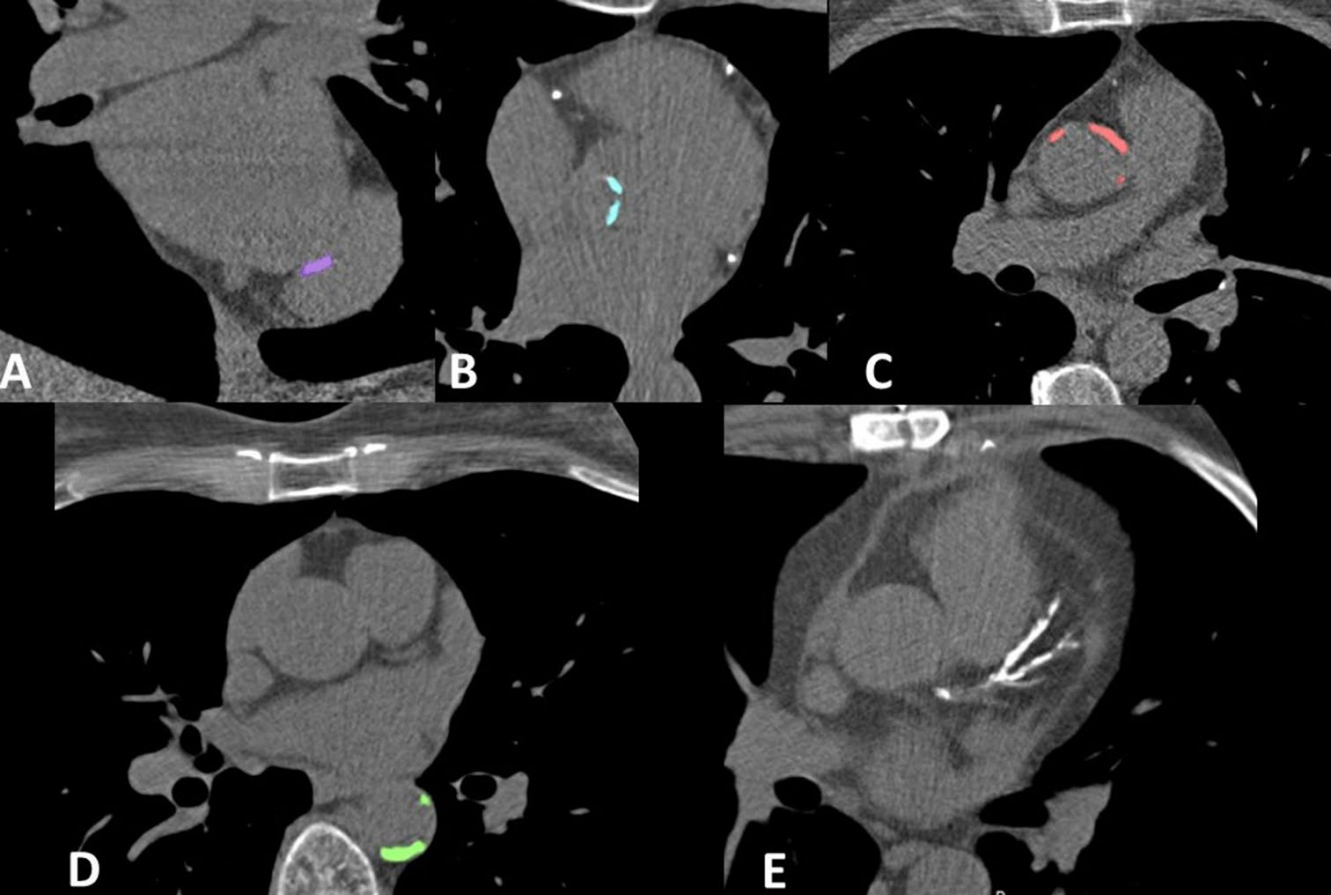
Examples of calcification as shown in the calcification scoring program. **a** Mitral annulus calcification. **b** Aortic valve calcification. **c** Ascending aorta calcification. **d** Descending aorta calcification. **e** Coronary artery calcification

Images were acquired using a 256-slice MDCT-scanner (iCT, Philips Healthcare, the Netherlands). Supplemental methods provide detailed information on the CT-scan protocol and image analysis. Non-contrast enhanced cardiac CT-scan, as well as coronary CT angiography images were acquired. Scoring of calcification spots was performed on the non-contrast enhanced cardiac CT images visualizing heart base to the pulmonary artery bifurcation. Lesions were identified by a single observer who was trained by an experienced radiologist and blinded for patient characteristics and patient outcomes. CAC was scored using the Agatston method [17]. Calcifications on heart valves and in the thoracic aorta were quantified using a pseudo-mass score, calculated by multiplying the mean calcium HU value by the region of interest (ROI) volume for every lesion, and summing up the scores of all the lesions. The thoracic aorta calcium score was comprised of the sum of the calcium scores of ascending and descending aorta. The heart valve calcium score consisted of the sum of the aortic valve and mitral annulus calcium scores. Figure 1 shows examples of calcification as shown in the calcification scoring program. More detailed description of CT-scan protocol and image analysis is described in Supplemental methods.

### Incident cardiovascular events or cardiovascular interventions

During follow-up, participants received biannual questionnaires to gain information on occurrence of recurrent cardiovascular disease, bleeding events, incident diabetes, end stage renal disease, and hospitalizations for cardiovascular interventions. Additional information was gathered by acquiring data from hospitals and general practitioners. All incident major cardiovascular events were independently judged by three physicians from an endpoint committee and conflicting classifications were resolved in consensus. Experienced research nurses adjudicated all cardiovascular interventions. Outcome of the current study was MACE+ , a combined endpoint of recurrent MACE and cardiovascular interventions. MACE was defined as non-fatal myocardial infarction, non-fatal stroke or vascular death. Cardiovascular interventions included percutaneous or surgical revascularization interventions, including carotid endarterectomy (CEA), percutaneous coronary intervention (PCI), coronary artery bypass grafting (CABG), major amputations, and peripheral artery stenting, angioplasty or bypass. Outcome definitions are described in detail in Supplemental Table S1. Patients were included in the study based on a cardiovascular event. If a procedure had already been planned in response to this cardiovascular event, this intervention was not counted as a recurrent event.

### Predictor selection and data preparation

Predictors were selected based on presence in both the original 10-year SMART-risk score [14] and the lifetime SMART-REACH risk score[15] (Supplemental Table S2). These risk models were previously developed and externally validated to estimate the risk of recurrent cardiovascular events in patients with clinically manifest CVD [13–15]. Number of locations of vascular disease was limited to two categories (instead of three); 1 or > 1 due to the low number of patients with > 2 locations of vascular disease (N = 6), resulting in the following eight predictors: age, sex, current smoking (yes/no), history of diabetes mellitus (yes/no), systolic blood pressure (mmHg), total cholesterol (mmol/L), creatinine (mmol/L), and > 1 location of vascular disease (yes/no); coronary heart disease (CHD), cerebrovascular disease (CeVD), or peripheral artery disease (PAD)). No missing data was observed for the predictor variables.

### Development of the prediction model with and without calcium scores

Cox proportional hazards models were developed for the combined outcome MACE+ , including the pre-specified predictors in the model. Too few events were observed for recurrent MACE specifically (N = 15) to perform reliable analysis. Coronary artery, thoracic aorta, and heart valve calcium scores were added to the models separately and combined, resulting in five models: (I) clinical predictors, no calcium scores (reference model), (II) model I+CAC score, (III) model I+TAC score, (IV) model I+heart valve calcium score (aortic valve and mitral annulus), (V) model I+CAC+TAC+heart valve scores. As there were only 8 competing events (non-CVD death) during follow-up, a competing risk adjusted model [18] was not considered necessary. Continuous predictors, including the cardiovascular calcium scores, were truncated at the 1st and 99th percentile to limit the effect of outliers [19].

Bootstrapping was implemented to correct for optimism; a preferred method above split-sample especially considering the relatively small dataset and limited number of events [20]. First, models were fitted on the full original data. Second, 1000 random bootstrap samples were drawn with replacement from the original dataset and models were refitted on each bootstrap sample. For every bootstrap sample, the difference between the performance of the bootstrap model in the bootstrap sample and the performance of the bootstrap model in the original data was determined. The average difference represented the average optimism of the models and was used to shrink model coefficients. In the original model as well as in the bootstrap models fitted in each separate bootstrap sample, linearity of the association between continuous predictors and the outcome variable was assessed by comparing Akaike’s Information Criterion (AIC) [19] of a linear, squared, and log transformation of the variable. Variables were transformed appropriately to improve robustness of the model. Proportional hazards assumptions were assessed in the original model visually by plotting the scaled Schoenfeld residuals against follow-up time and no violations were observed.

### Comparison of models with and without calcium scores

Prognostic performances of the five models was evaluated following previously recommended steps [21]. First, global model fit was compared by assessing the AIC of the models with and without calcium scores. Secondly, model validation was performed by assessment of various model performance measures. The validation was performed for outcome data from 4 years of follow-up (approximation of 75% percentile of follow-up duration). The calibration plots of predicted versus observed risk were compared and the c-statistic for discrimination. C-statistics were adjusted to account for optimism by assessing model performance in 1000 bootstrap samples, with confidence intervals based on the percentile method. As c-statistics usually lack power to compare models, an additional risk reclassification test is recommended [21, 22]. A categorical NRI for survival data with right censoring was calculated (R package nricens), with predetermined 4-year risk categories. These risk categories were < 8%, 8–13%, 13–18%, and > 18%, based on interpolation by linearly adapting 10-year risks (< 20%, 20–30%, 30–40%, and > 40%) to 4-year risks. As no risk thresholds for preventive treatment are known for secondary prevention, or for the combined outcome MACE + specifically, the risk difference based NRI was additionally calculated. The cut-off value was set at 0.02, meaning that only differences in predicted probability of ≥ 2% contributed (corresponding to a 10-year risk of 5%). Detailed methodological description of the NRI analysis is given in Supplemental methods.

Model development and validation was additionally assessed for models with presence or absence (calcium score < 10) of calcification instead of continuous scores for comparison. All analyses were performed in R-Statistic Programming (version 3.5.1).

## Results

Baseline characteristics are shown in Table 1. Mean age was 58 (SD 9) years, and prevalence of males was 77%. History of coronary artery disease was the most prevalent type of vascular disease at baseline (72%). Median (range) calcium scores were 202 (0–3941) for coronary arteries (Agatston score), and 2 (0–1820) for thoracic aorta and 1 (0–838) for heart valves (pseudo-mass score). During a median follow-up time of 3.43 years (interquartile range (IQR) 2.28–4.74) 15 recurrent cardiovascular events occurred; 6 non-fatal strokes, 7 non-fatal myocardial infarctions, and 2 vascular deaths. For the combined endpoint MACE+ (counting the first event), 77 events were observed; 5 non-fatal strokes, 6 non-fatal myocardial infarctions, 2 carotid artery interventions, 49 cardiac interventions (5 CABG and 44 PCI), 14 peripheral artery and abdominal aortic interventions, and 1 vascular death.

**Table 1:** Baseline characteristics

	Total, N = 567
Male, n (%)	441 (77%)
Age (years)*	58 ± 9
Current smoking, n (%)	143 (25%)
Number of pack-years*	9 (0–24)
Medical history	
Cerebrovascular disease (CeVD), n (%)	165 (29%)
Coronary heart disease (CHD), n (%)	408 (72%)
Peripheral artery disease (PAD), n (%)	29 (5%)
Multifocal vascular disease (eg. CHD and PAD), n (%)	52 (9%)
Diabetes mellitus, n (%)	63 (11%)
Physical examination and laboratory measurements	
Body Mass Index (kg/m^2^)*	27 ± 4
Systolic blood pressure (mmHg)*	129 ± 15
Diastolic blood pressure (mmHg)*	78 ± 9
Triglycerides (mmol/L)*	1.3 (1.0–1.8)
Total cholesterol (mmol/L)*	4.4 ± 1.1
HDL-cholesterol (mmol/L)*	1.2 (1.0–1.4)
Hs-CRP (mg/L)*	1.4 (0.7–3.3)
eGFR (CKD-EPI, mL/min/1.73m^2^)*	89 ± 13
Medication	
Lipid lowering medication, n (%)	482 (85%)
Blood pressure lowering agents, n (%)	457 (81%)
Anti-platelet therapy, n (%)	499 (88%)
Anti-coagulants, n (%)	37 (7%)
Cardiovascular calcium scores	
Thoracic aorta calcium score^†^	2 (0–1820)
Coronary artery calcium score^†^	202 (0–3941)
Aortic valve and mitral annulus calcium score^†^	1 (0–838)

*Data are means ± SD or median (interquartile range). eGFR = estimated glomerular filtration rate

^†^Coronary artery calcium score is Agatston score. Thoracic and valve calcium scores are pseudo mass scores. Median (range) is given

### Development of models with and without calcium scores

AIC of the model without calcium score was 851. AIC was lower, showing a better model fit, for model II with CAC score (AIC 846) and model V with all calcium scores (AIC 848). Thoracic aorta calcium and valve calcium scores did not improve model fit according to the AIC (AIC 853 for model III and AIC 853 for model IV respectively). Model coefficients and model formulas of models I, II, III, IV, and V are shown in Supplemental Tables S3 and S4. The truncated and log-transformed CAC score was statistically significantly related to the outcome MACE+ (HR 1.53; 95%CI 1.11–2.14). The truncated (and log-transformed for the valve calcium score) score of heart valves and thoracic aorta showed no statistically significant relation with the outcome.

### Discrimination and calibration

**Fig. 2:**
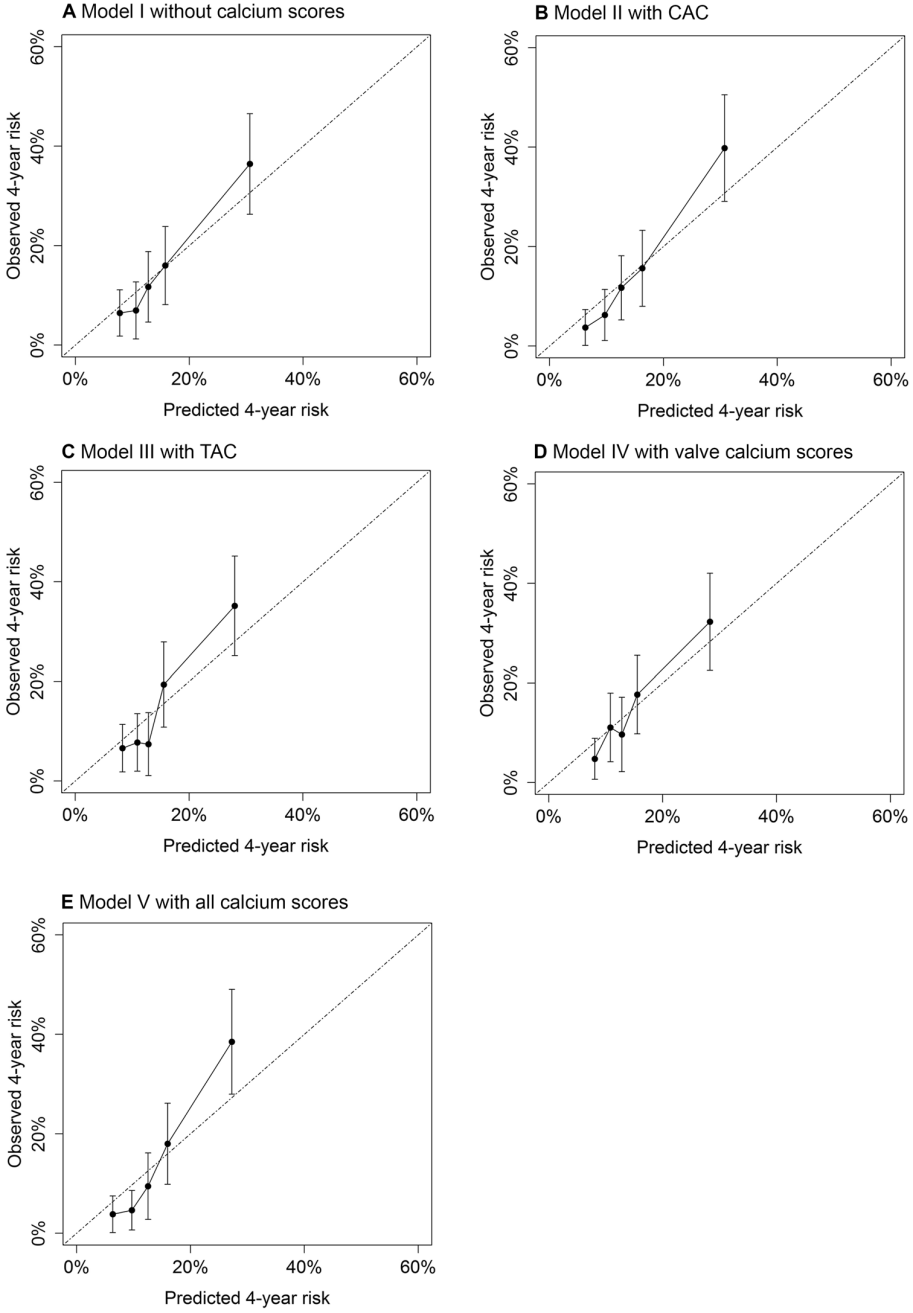
Calibration plots of models without and with calcium scores for the prediction of MACE+

Performance of the models was assessed by comparing calibration and discrimination. Figure 2 shows calibration plots of the predicted versus observed 4-year risk of MACE + for the different models. Model I without calcium scores (Fig. 2a) shows good calibration. Models with CAC (Fig. 2b), TAC (Fig. 2c), valve calcium (Fig. 2d), and all calcium scores (Fig. 2e) show similar calibration and no clear improvement compared to the calibration of model I. Optimism corrected c-statistics were 0.65; 95%CI 0.59–0.72 for model I without calcium scores, 0.67; 95%CI 0.61–0.73 for model II with CAC, 0.65; 95%CI 0.59–0.72 for model III with TAC, 0.65; 95%CI 0.59–0.72 for model IV with valve calcium, and 0.68; 95%CI 0.62–0.74 for model V with all calcium scores.

### Net reclassification index

**Fig. 3:**
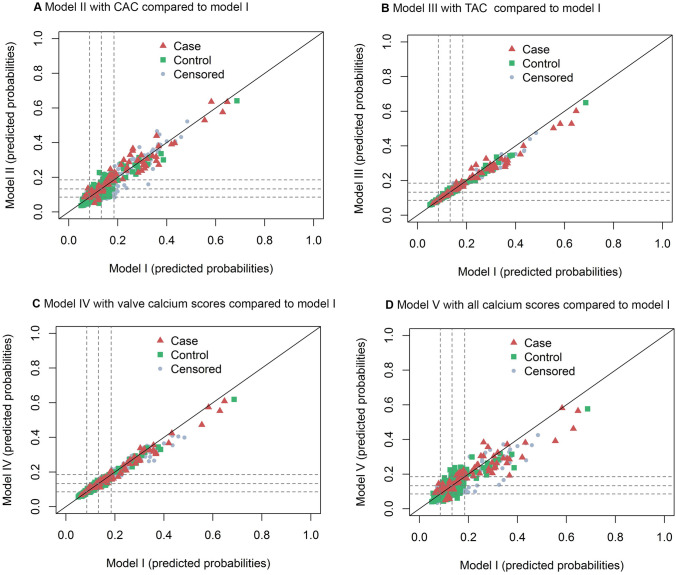
Predicted probabilities for patients with an event and patients without an event by models with calcium scores compared to model I without calcium scores for the prediction of MACE+ . The diagonal line added to the plot indicates no change in the predicted probabilities. If the expanded prediction model improved reclassification, events will lie above the diagonal (higher predicted probability with the new model) and will have switched to a higher risk category, whereas controls will appear below the diagonal (lower predicted probability with the new model) and will have switched to a lower risk category. The dotted lines represent the 4-year risk thresholds: 9%, 13%, and 18%

Model II with addition of the CAC score generally reclassified patients correctly to a higher or lower risk category, according to the categorical NRI: 15.24%; 95%CI 0.59–29.39 (Table 2). Especially in patients without an event, model II reclassified patients to a lower risk category (reclassification index 8.93%; 95%CI 2.98–15.03 in the group of patients without event). Model V with all calcium scores also improved risk reclassification, as shown by a categorical NRI of 20.00%; 95%CI 5.59–34.92. Models III-V with addition of the TAC score, valve calcium score or all calcium scores did not improve risk category classification. For the risk difference based NRI, similar results were observed with improvement in risk classification by model II with the CAC score (24.76%; 95%CI 5.10–43.60), but no improvement for models III-V) (Supplemental Table S4). Figure 3 shows scatterplots of predicted probabilities based on the original model versus the predicted probabilities based on the expanded models including calcium scores, with symbols for patients with an event, patients without an event, and censored subjects. Model II with the CAC score and model V with all calcium scores show differences in predicted risks comparing the expanded model with the reference model without calcium scores (Fig. 3a and d). For model III with the TAC and IV with the valve calcium score (Fig. 3b and c), the expanded models hardly changed risk predictions, as both patients with and patients without an event are situated along the diagonal.

**Table 2:** Categorical net reclassification index comparing models with calcium scores to model I without calcium scores for the prediction of MACE +

	Categorical reclassification index* (%)		
	With event (95% CI)	Without event (95% CI)	Net (95% CI)
**Model 1** No scores	ref	ref	ref
**Model II** CAC score	6.31 (− 6.23 to 18.56)	**8.93** **(2.87–15.03)**	**15.24** **(0.59–29.39)**
**Model III** TAC score	0.10 (− 5.44 to 5.92)	− **3.45** **(**− **6.73 to **− **0.19)**	− 3.34 (− 9.97 to 3.95)
**Model IV** Valve scores	− 5.29 (− 12.54 to 1.06)	1.21 (− 2.60 to 4.76)	− 4.08 (− 12.35 to 3.39)
**Model V** All scores	9.25 (− 4.60 to 23.31)	**10.76** **(4.93–23.31)**	**20.00** **(5.59–34.92)**

Bold values are statistically significant

*Categories for the categorical were based on 10-year risk categories < 20%, 20–30%, 30–40%, and > 40% translated to 4-year risks: < 9%, 9–13%, 13–18%, > 18%

Performance of models with addition of a calcification predictor indicating presence or absence of calcium (instead of continuous scores) showed similar results (model coefficients, calibration plots, c-statistics, and NRI in Supplemental Table S5, Fig. S1, and Table S6). Sensitivity analyses with a cut-off value of > 0 for presence of calcium did not change the results.

## Discussion

The present study shows that in patients with established cardiovascular disease, addition of the CAC score to a prediction model with classical atherosclerotic risk factors for estimating the risk of MACE+ , provides similar calibration and discrimination, and improves global model fit and risk classification for 4-year risk predictions. Addition of the TAC or heart valve calcium score did not improve measures of model performance.

Extra-coronary thoracic cardiovascular calcification scores, including thoracic aorta and heart valves, did not improve risk predictions in the current study, in accordance with previous studies in patients without established cardiovascular disease [11, 12]. Addition of the CAC score did improve risk predictions in the current study, in terms of global model fit and risk reclassification. Similar calibration and c-statistics were observed for the model with the CAC score in comparison to the model with only traditional risk factors. However, c-statistics often lack statistical power to compare models, and conclusions should not be solely based on this model performance measure [21, 22]. Although calibration, an important measure for prognostic risk model performance, [21] was similar, the NRI showed that addition of the CAC score to a prediction model with traditional risk factors correctly reclassified patients to a higher or lower risk category. Therefore, the CAC score was considered of additional prognostic value for the prediction of MACE+ in patients with established cardiovascular disease. These results are also in accordance with previous studies in apparently healthy people where addition of the CAC score to models with classical risk factors was found to improve model performance [7, 8, 23–27]. Furthermore, a relation between the CAC score and the combined endpoint MACE+ in patients with stable CVD was previously observed, with a HR of 1.35; 95%CI 1.15–1.58 (per SD higher calcium score) [28], and between CAC > 0 and MACE in patients with suspected CHD with a pooled relative risk ratio of 5.71; 95%CI 3.98–8.19 [3].

Coronary artery calcification can be regarded as a measure of total plaque burden, [6] and in that capacity calcium scores will provide additional prognostic value for the prediction of (recurrent) cardiovascular events. The process of coronary artery calcification is thought to act as a plaque stabilizer, [5] as large dense calcification spots of more than 400 Hounsfield Units (HU) are commonly associated with stable plaques and microcalcifications of lower density with instable, vulnerable plaques [5, 29] Furthermore, long term and high dose statin use is thought to accelerate coronary plaque calcification without leading to more frequent cardiovascular events, [30] suggesting that CAC increase under statin treatment represents plaque stabilization rather than plaque expansion [30–32]. As was previously shown in patients without established CVD, [33–36] markers of calcification morphology or stenosis severity, undistinguished by the CAC score, potentially provide additional prognostic value for risk prediction beyond the CAC score in patients with established CVD, and this will be investigated in future work.

Primary prevention guidelines recommend to use available CAC scores in patients with predicted risks around 5% or 10% risk factor treatment thresholds for risk reclassification [10]. Furthermore, the ongoing ‘risk or benefit in screening for cardiovascular diseases’ (ROBINSCA) trial, a large-scale population-based cardiovascular disease screening trial, is investigating the impact of CAC imaging and subsequent preventive treatment on CVD morbidity and mortality in apparently healthy people [37, 38]. As the present study showed that the CAC score improves risk estimations of MACE+ in patients with established cardiovascular disease, implementation of available CAC scores in risk prediction could be recommended. Particularly in patients with established CVD, CAC scores are often available as CT-imaging of the chest is often performed for various diagnostic indications in these patients. Although no risk thresholds for preventive treatment are available for secondary prevention, accurate risk predictions could lead to justified treatment intensification or downgrading. Novel and costly therapies are available, such as PCSK9 inhibitors, [39] and new antithrombotic treatment schemes, such as dual antiplatelet therapy or adding rivaroxaban to aspirin (dual pathway inhibition), [40] aiming to reduce cardiovascular disease risk. Accurate risk predictions are needed to distinguish patients with the highest risk that will benefit the most from these novel therapies or to identify patients with the lowest risk to possibly refrain from intensifying preventive therapy. Currently, the SMART risk score [14] and the SMART-REACH model [15] are the standard for 10-year and lifetime predictions, respectively, of recurrent cardiovascular events in patients with established cardiovascular disease. Although these models performed well in external validation, [13, 15, 41] further improvement could enhance prediction accuracy. In future studies, risk prediction models could be developed with addition of the CAC score as extensions to the SMART-risk and SMART-REACH model, for patients with an available CAC score. These models could estimate the risk of MACE+ , and potentially the CAC score is also of added prognostic value for the prediction of MACE specifically. Since the simple model with CAC absence or presence performed similarly compared to the model with the continuous CAC score, it might be considered to develop models with the continuous CAC score as well as a simple presence or absence score, in order to benefit the most of available information for cardiovascular risk prediction in patients with established cardiovascular disease. Effects of risk prediction modeling including CAC scores on preventive treatment strategies in patients with established CVD and subsequent mortality and morbidity should be investigated in future studies.

The present study has several strengths, including the cohort of patients with established cardiovascular disease with available cardiovascular calcium scores and follow-up data. Furthermore, several model performance measures were evaluated, including discrimination, calibration, and NRI. Limitations should be considered and include the limited length of follow-up. Therefore, validation of the models could only be performed for 4-years of follow-up. Furthermore, due to the limited number of events, reliable analyses of specific recurrent cardiovascular events (recurrent MACE specifically, stroke or coronary heart disease), or subgroup analyses could not be performed. In patients with coronary artery stents, arterial segments with stents were excluded, and total CAC scores might thus be underestimated in these patients. Subgroup analyses in patients with coronary heart, cerebrovascular or peripheral artery disease specifically would strengthen generalizability to all patients with established cardiovascular disease. As the number of events (N = 77) is limited, and does not reach the recommended minimum number of 100 events for validation of a prediction model [42, 43], the results of the current study are preliminary findings for a population with established cardiovascular disease, for whom to our knowledge no previous studies were specifically performed to assess the added prognostic value of calcium scores. Additionally, calcification lesions were not assessed by a second observer. However, all extra-coronary lesions were scored by one individual, thereby limiting between-image variability. Furthermore, patients with reduced renal function were excluded, and results of the current study cannot be generalized to patients with established CVD and reduced renal function. Lastly, models were internally validated potentially leading to optimism, where external validation would be preferred. However, discrimination and calibration were adjusted for optimism by bootstrapping, limiting this effect [44].

In conclusion, in patients with established CVD, addition of the CAC score improved performance of a risk prediction model based on classical risk factors, for the prediction of the combined endpoint MACE+ . Addition of the TAC or heart valve score did not improve risk predictions.

## Supplementary Information

Below is the link to the electronic supplementary material.

Supplementary Information 1 (DOCX 1983 kb)

## Data Availability

The data underlying this article will be shared on reasonable request to the corresponding author.

## Abbreviations

AIC: Akaike’s Information Criterion
CABG: Coronary artery bypass grafting
CAC: Coronary artery calcium
CEA: Carotid endarterectomy
CeVD: Cerebrovascular disease
CHD: Coronary heart disease
CT: Computed tomography
CTA: Computed tomography angiograhpy
CVD: Cardiovascular disease
eGFR: Estimated glomerular filtration rate
HU: Hounsfield units
MACE: Major cardiovascular events
NRI: Net reclassification index
PAD: Peripheral artery disease
PCI: Percutaneous coronary intervention
TAC: Thoracic aorta calcium
UCC-SMART: Utrecht Cardiovascular Cohort-Second Manifestation of ARTerial disease
