# Supplementary material for: Added value of cardiovascular calcifications for prediction of recurrent cardiovascular events and cardiovascular interventions in patients with established cardiovascular disease
Source: Int J Cardiovasc Imaging. 2021 Feb 12;37(6):2051–61. doi: 10.1007/s10554-021-02164-9 (PMC8255266; doi:10.1007/s10554-021-02164-9)
Supplement: Supplementary file 1 — Supplementary Information 1 (DOCX 1983 kb) [file 10554_2021_2164_MOESM1_ESM.docx]

**Supplemental Data**

**Added value of cardiovascular calcifications for prediction of recurrent cardiovascular events and cardiovascular interventions in patients with established cardiovascular disease**

*Authors: Cilie C. van ‘t Klooster, Yolanda van der Graaf, Hendrik M. Nathoe, Michiel L. Bots, Gert Jan de Borst, Frank L.J. Visseren, Tim Leiner*

**Table of content**

[Supplemental methods 3](#_Toc40197233)

[Supplemental Table S1. Predictor and outcome definitions in the UCC-SMART cohort 5](#_Toc40197234)

[Supplemental table S2. Predictor selection based on presence in both SMART-REACH model and SMART-risk score 7](#_Toc40197235)

[Supplemental table S3. Hazard ratios, 95% confidence intervals, and shrinkage factor of models with and without calcium scores 8](#_Toc40197236)

[Supplemental table S4. Risk difference based net reclassification index comparing models with calcium scores to model I without calcium scores for the prediction of MACE+ 10](#_Toc40197237)

[Supplemental table S5. Hazard ratios, 95% confidence intervals, and shrinkage factor of models with and without calcium presence/absence for the prediction of MACE+ 11](#_Toc40197238)

[Supplemental figure S1. Calibration plots of models with calcium presence/absence for the prediction of MACE+ 13](#_Toc40197239)

[Supplemental table S6. C-statistics and net reclassification index for models with and without calcium presence/absence as predictor for MACE+ 14](#_Toc40197240)

# Supplemental methods

CT-scan protocol

Non-contrast enhanced cardiac CT-scans were ECG gated with a standard tube voltage of 120 peak kilovoltage (kVp). To achieve most optimal imaging for the coronary CTA, patients whose heart rate exceeded 70 bpm were given beta-adrenergic blocking medication. A weight-dependent bolus of 70-90 ml iodine contrast was injected. Coronary CT angiography was performed using prospectively triggered protocols synchronized to the diastolic resting phase (78% of the RR-interval). In patients in whom the heart rate could not be lowered to 70 beats or less minute, or in cases of irregular heart rate retrospective gating was used.

Image analysis

For 2 patients coronary artery calcification score was missing. For 7 patients, a non-contrast enhanced cardiac scan was absent, due to missing thin slice reconstruction, interfering artefacts, or technical problems. Patients with aortic valve replacement (n=9) or mitral valve replacement (n=6) were excluded from the analysis with heart valve calcification scores. Therefore, final study population numbers were 565 for development and validation of model II (with CAC), 560 for model III (with TAC), 546 for model IV (with valve calcium scores), and 545 for model V (with all calcium scores).

Thin slice reconstructions were created for scoring thoracic aorta and cardiac valve calcification with a slice thickness of 0.9 mm at 0.45 mm increment. For coronary arteries a slice thickness of 3.0 mm at 3.0 mm increment was used according to Agatston guidelines.(1)

Calcification lesions were identified by the observer and the dedicated software (iX Viewer, ImageSciences Institute, Utrecht, the Netherlands) automatically selected calcifications across consecutive slices. The automatic selection was inspected per slice and manually corrected if necessary, to prevent overestimation. Calcification in the aortic root was considered part of the aortic valve.(2) If a calcification lesion occupied multiple anatomical locations, it was scored according to its predominant location.

For the selection of calcification on the non-contrast enhanced scans a standard threshold of 130 Hounsfields Units (HU) was implemented.

CAC was scored using the Agatston method.(1) Calcifications on heart valves and in the thoracic aorta were quantified using a pseudo-mass score, calculated by multiplying the mean calcium HU value by the region of interest (ROI) volume for every lesion, and summing up the scores of all the lesions.

Data analysis

*Development of the prediction model with and without calcium scores*

As there were only 8 competing events (non-CVD death) during follow-up, a competing risk adjusted model(3) was not considered necessary. First, models were fitted on the full original data. Second, 1000 random bootstrap samples were drawn with replacement from the original dataset and models were refitted on each bootstrap sample. For every bootstrap sample, the difference between the performance of the bootstrap model in the bootstrap sample and the performance of the bootstrap model in the original data was determined. The average difference represented the average optimism of the models and was used to shrink model coefficients. In the original model as well as in the bootstrap models fitted in each separate bootstrap sample, linearity of the association between continuous predictors and the outcome variable was assessed by comparing Akaike’s Information Criterion (AIC)*(4)* of a linear, squared, and log transformation of the variable. Variables were transformed appropriately to improve robustness of the model.

*Net reclassification index*

The continuous net reclassification index (NRI) is calculated by assessing the change in predicted probability for cases and controls comparing the base model to models with an additional predictor, and is defined as: NRI = P(up|event) - P(down|event) + P(down|nonevent) - P(up|event). Originally, the NRI was developed for binary outcome and did not handle censoring.(5, 6) A prospective form of NRI has been proposed, allowing for NRI calculations in survival data with right censoring.(7) The new formulation of the NRI can be interpreted as a measure of event rate increase among those who are reclassified upwards, and event rate decrease among those who are reclassified downwards, with event rates estimated by the Kaplan-Meier approach.(7) The reclassification can graphically be displayed by plotting predicted probabilities based on the original model versus the predicted probabilities based on the extended model, with symbols for cases and controls. The diagonal line added to the plot indicates no change in the predicted probabilities. If the extended prediction model improved reclassification, events will lie above the diagonal (higher predicted probability with the new model), whereas controls will appear below the diagonal (lower predicted probability with the new model).(7, 8)

# Supplemental Table S1. Predictor and outcome definitions in the UCC-SMART cohort

1. Definition of baseline characteristics (9, 10)

| **Age** | Years, reported by physician/patient |
| --- | --- |
| **Sex** | Male/female, reported by physician/patient |
| **Current smoking** | Current vs never/former, reported by patient |
| **History of diabetes mellitus** | Either referral diagnosis, self-reported, or a known history of diabetes mellitus at the time of enrolment or a fasting blood glucose ≥7 mmol/L. |
| **Systolic blood pressure** | mmHg. Measured directly after informed consent. Mean of two office blood pressure measurements. |
| **Total cholesterol** | mmol/L. Measured in fasting venous sample using commercial enzymatic dry chemistry kits (Johnson and Johnson) |
| **Creatinine** | µmol/L. Measured in fasting venous sample using commercial enzymatic dry chemistry kits (Johnson and Johnson). |
| **History of peripheral artery disease** | Symptomatic and documented obstruction of distal arteries of the leg and/or a (recent) history of revascularization of the leg (percutaneous transluminal angioplasty or bypass surgery) or amputation |
| **History of coronary heart disease** | Documented angina pectoris, myocardial infarction or coronary revascularization (coronary bypass surgery or coronary angioplasty) |
| **History of cerebrovascular disease** | Documented TIA, cerebral infarction, amourosis fugax or retinal infarction, or a history of carotid surgery |

1. Definition of recurrent cardiovascular disease (9, 10)

| **Non-fatal myocardial infarction** | At least two of the following: 1. Chest pain for at least 20 minutes, not disappearing after administration of nitrates; 2. ST-elevation >1 mm in two following leads or a left bundle branch block on the ECG; 3. CK elevation of at least two times the normal value of CK and a MB-fraction >5% of the total CK. |
| --- | --- |
| **Non-fatal stroke** | Relevant clinical features which have caused an increase in handicap of at least one grade on the modified Rankin scale,  accompanied by fresh infarct or hemorrhage on a repeat CT scan |
| **Vascular death** | Sudden death (unexpected cardiac death occurring within 1 hour after onset of symptoms, or within 24 hours given convincing  circumstantial evidence) or death from stroke, myocardial  infarction, congestive heart failure, rupture of abdominal aortic aneurysm, or from other cause, i.e. sepsis following stent placement. |

1. Definition of cardiovascular intervention (9, 10)*

| **Heart** | Percutaneous coronary intervention or coronary artery bypass surgery |
| --- | --- |
| **Carotid artery or intracranial arteries** | Stent, angioplasty, (thrombo)endarterectomy, bypass surgery |
| **Peripheral** | Stent or graft (endovascular or open surgery), angioplasty, bypass surgery or (thrombo)endarterectomy. Major amputation (excluding toe or forefoot amputation(11)) due to arterial ischemia. Other intervention due to ischemia (eg emergency laparotomy due to intestinal ischemia, urokinase treatment, surgical correction of endoleak after EVAR, nephrectomy due to atherosclerotic cause, kidney transplantation) |
| * Patients who received a vascular intervention in response to a cardiovascular disease event, are classified according to the cardiovascular disease event | |

# Supplemental table S2. Predictor selection based on presence in both SMART-REACH model and SMART-risk score

| **SMART-REACH model(10)** | **SMART-risk score(12)** | **Final selection for current study** |
| --- | --- | --- |
| Age (years) | Age (years) | Age (years) |
| Sex (male/female) | Sex (male/female) | Sex (male/female) |
| Current smoking (yes/no) | Current smoking (yes/no) | Current smoking (yes/no) |
| Diabetes mellitus (yes/no) | Diabetes mellitus (yes/no) | Diabetes mellitus (yes/no) |
| Number of locations of vascular disease (1, 2, or 3) | Coronary heart disease, cerebrovascular disease, peripheral artery disease, abdominal aorta aneurysm as 4 separate predictors | >1 location of vascular disease |
| Systolic blood pressure (mmHg) | Systolic blood pressure (mmHg) | Systolic blood pressure (mmHg) |
| Total cholesterol (mmol/L) | Total cholesterol (mmol/L) | Total cholesterol (mmol/L) |
| Creatinine (µmol/L) | Estimated glomerular filtration rate (ml/min/1.73 m^2^) | Creatinine (µmol/L) |
| Atrial fibrillation (yes/no) |  |  |
| Congestive heart failure (yes/no)* |  |  |
|  | HDL cholesterol (mmol/L) |  |
|  | C-reactive protein (mg/L) |  |
|  | Years since first vascular event |  |
| * Not available in UCC-SMART data | | |

# Supplemental table S3. Hazard ratios, 95% confidence intervals, and shrinkage factor of models with and without calcium scores

| **A** Model I (No calcium scores) |  |  | |  | | |  |
| --- | --- | --- | --- | --- | --- | --- | --- |
|  | **Coefficient** | **HR** | | **95% CI lower limit** | | | **95% CI upper limit** |
| Age | 0.0495 | 1.0507 | | 0.8167 | | | 1.3517 |
| Creatinine | -0.0675 | 0.9347 | | 0.7231 | | | 1.2082 |
| Sex | 0.7159 | 2.0461 | | 0.9596 | | | 4.3624 |
| Systolic blood pressure | 0.2138 | 1.2384 | | 0.9927 | | | 1.5448 |
| Total cholesterol | 0.1631 | 1.1771 | | 0.9182 | | | 1.5091 |
| Current smoking | 0.0567 | 1.0584 | | 0.6208 | | | 1.8043 |
| Diabetes mellitus | 1.0864 | 2.9636 | | 1.7546 | | | 5.0056 |
| >1 location of vascular disease | 1.1180 | 3.0588 | | 1.7672 | | | 5.2943 |
| Shrinkage factor 0.8278. For continuous predictors, hazard ratios per 1SD are given | | | | | | | |
| **B** Model II (coronary artery calcium score) | | | | | | | |
|  | **Coefficient** | **HR** | | **95% CI lower limit** | | **95% CI upper limit** | |
| Age | -0.1144 | 0.8919 | | 0.6706 | | 1.1861 | |
| Creatinine | -0.0321 | 0.9684 | | 0.7510 | | 1.2488 | |
| Sex | 0.4839 | 1.6224 | | 0.7499 | | 3.5101 | |
| Systolic blood pressure | 0.2012 | 1.2229 | | 0.9786 | | 1.5283 | |
| Total cholesterol | 0.1937 | 1.2137 | | 0.9450 | | 1.5589 | |
| Current smoking | 0.0388 | 1.0396 | | 0.6091 | | 1.7743 | |
| Diabetes mellitus | 1.0787 | 2.9409 | | 1.7453 | | 4.9556 | |
| >1 location of vascular disease | 0.9636 | 2.6211 | | 1.5003 | | 4.5792 | |
| log(coronary artery calcium score) | 0.4276 | 1.5336 | | 1.1006 | | 2.1370 | |
| Shrinkage factor 0.8037. For continuous predictors, hazard ratios per 1SD are given | | | | | | | |
| **C** Model III (thoracic aorta calcium score) | | | | |  | | |
|  | **Coefficient** | **HR** | **95% CI lower limit** | | **95% CI upper limit** | | |
| Age | 0.0212 | 1.0214 | 0.7802 | | 1.3372 | | |
| Creatinine | -0.1183 | 0.8884 | 0.6825 | | 1.1564 | | |
| Sex | 0.7554 | 2.1284 | 0.9968 | | 4.5448 | | |
| Systolic blood pressure | 0.1764 | 1.1929 | 0.9464 | | 1.5036 | | |
| Total cholesterol | 0.1488 | 1.1604 | 0.9023 | | 1.4924 | | |
| Current smoking | 0.0794 | 1.0827 | 0.6343 | | 1.8479 | | |
| Diabetes mellitus | 0.9670 | 2.6300 | 1.5097 | | 4.5816 | | |
| >1 location of vascular disease | 1.0823 | 2.9516 | 1.6711 | | 5.2133 | | |
| Thoracic aorta calcium score | 0.0660 | 1.0683 | 0.8824 | | 1.2932 | | |
| Shrinkage factor 0.7704. For continuous predictors, hazard ratios per 1SD are given | | | | | | | |

| **D** Model IV (valve calcium scores) | | |  | |  |
| --- | --- | --- | --- | --- | --- |
|  | **Coefficient** | **HR** | **95% CI lower limit** | | **95% CI upper limit** |
| Age | 0.1229 | 1.1308 | 0.8407 | | 1.5209 |
| Creatinine | -0.1246 | 0.8829 | 0.6752 | | 1.1544 |
| Sex | 0.7873 | 2.1974 | 1.0201 | | 4.7336 |
| Systolic blood pressure | 0.1646 | 1.1789 | 0.9322 | | 1.4910 |
| Total cholesterol | 0.1555 | 1.1683 | 0.9054 | | 1.5076 |
| Current smoking | 0.0588 | 1.0606 | 0.6097 | | 1.8449 |
| Diabetes mellitus | 1.0888 | 2.9707 | 1.7055 | | 5.1744 |
| >1 location of vascular disease | 1.1771 | 3.2450 | 1.7941 | | 5.8692 |
| log(valves calcium score) | -0.1111 | 0.8948 | 0.6847 | | 1.1694 |
| Shrinkage factor 0.7791. For continuous predictors, hazard ratios per 1SD are given | | | | | |
| **E** Model V (all calcium scores combined) | | | |  | |
|  | **Coefficient** | **HR** | **95% CI lower limit** | **95% CI upper limit** | |
| Age | -0.0292 | 0.9712 | 0.6972 | 1.3528 | |
| Creatinine | -0.0975 | 0.9071 | 0.6948 | 1.1843 | |
| Sex | 0.5476 | 1.7291 | 0.7968 | 3.7519 | |
| Systolic blood pressure | 0.1517 | 1.1638 | 0.9168 | 1.4774 | |
| Total cholesterol | 0.1998 | 1.2212 | 0.9437 | 1.5801 | |
| Current smoking | 0.0506 | 1.0519 | 0.6028 | 1.8357 | |
| Diabetes mellitus | 1.0163 | 2.7631 | 1.5703 | 4.8618 | |
| >1 location of vascular disease | 0.9862 | 2.6809 | 1.4455 | 4.9722 | |
| log(valves calcium score) | -0.2045 | 0.8151 | 0.6178 | 1.0752 | |
| Thoracic aorta calcium score | 0.0544 | 1.0559 | 0.8614 | 1.2943 | |
| log(coronary artery calcium score) | 0.4953 | 1.6410 | 1.1580 | 2.3254 | |
| Shrinkage factor 0.7277. For continuous predictors, hazard ratios per 1SD are given | | | | | |

# Supplemental table S4. Risk difference based net reclassification index comparing models with calcium scores to model I without calcium scores for the prediction of MACE+

|  | **Risk difference based reclassification index (%)** | | |
| --- | --- | --- | --- |
|  | **With event**  **(95% CI)** | **Without event (95% CI)** | **Net**  **(95% CI)** |
| **Model 1**  No scores | ref | ref | ref |
| **Model II**  CAC score | 14.74  (-3.16-31.46) | **10.01**  **(2.82-17.07)** | **24.76**  **(5.10-43.60)** |
| **Model III**  TAC score | **-19.45**  **(-30.80- -7.73)** | **6.67**  **(3.59-9.77)** | **-12.78**  **(-24.33- -0.53)** |
| **Model IV**  Valve scores | **-22.04**  **(-35.64- -9.26)** | **5.99**  **(2.32-9.46)** | **-16.05**  **(-29.68- -2.55)** |
| **Model V**  All scores | -2.41  (-21.73-16.29) | **18.62**  **(11.38-25.09)** | 16.20  (-6.21-36.37) |
| A cut-off value of 2% was used for the risk difference based reclassification index | | | |

# Supplemental table S5. Hazard ratios, 95% confidence intervals, and shrinkage factor of models with and without calcium presence/absence for the prediction of MACE+

| **A** Model II (coronary artery calcium) | | |  |
| --- | --- | --- | --- |
|  | **HR** | **95% CI lower limit** | **95% CI upper limit** |
| Age | 0.9435 | 0.7200 | 1.2363 |
| Creatinine | 0.9585 | 0.7422 | 1.2377 |
| Sex | 1.7517 | 0.8132 | 3.7733 |
| Systolic blood pressure | 1.2042 | 0.9367 | 1.5480 |
| Total cholesterol | 1.0251 | 0.6005 | 1.7497 |
| Current smoking | 1.0251 | 0.6005 | 1.7497 |
| Diabetes mellitus | 2.8912 | 1.7168 | 4.8689 |
| >1 location of vascular disease | 2.8108 | 1.6210 | 4.8738 |
| Coronary artery calcium present | 2.6520 | 1.0846 | 6.4843 |
| Shrinkage factor 0.8067. For continuous predictors, hazard ratios per 1SD are given | | | |

| **B** Model III (thoracic aorta calcium) | | |  |
| --- | --- | --- | --- |
|  | **HR** | **95% CI lower limit** | **95% CI upper limit** |
| Age | 0.9992 | 0.7507 | 1.3300 |
| Creatinine | 0.8919 | 0.6872 | 1.1576 |
| Sex | 2.1915 | 1.0219 | 4.6994 |
| Systolic blood pressure | 1.1938 | 0.9492 | 1.5014 |
| Total cholesterol | 1.1481 | 0.8916 | 1.4783 |
| Current smoking | 1.0694 | 0.6255 | 1.8283 |
| Diabetes mellitus | 2.6396 | 1.5243 | 4.5710 |
| >1 location of vascular disease | 3.0354 | 1.7467 | 5.2751 |
| Thoracic aorta calcium present | 1.2514 | 0.7131 | 2.1961 |
| Shrinkage factor 0.7986. For continuous predictors, hazard ratios per 1SD are given | | | |

| **C** Model IV (valve calcium) | |  |  |
| --- | --- | --- | --- |
|  | **HR** | **95% CI lower limit** | **95% CI upper limit** |
| Age | 1.0968 | 0.8237 | 1.4605 |
| Creatinine | 0.8836 | 0.6763 | 1.1544 |
| Sex | 2.1698 | 1.0086 | 4.6679 |
| Systolic blood pressure | 1.1785 | 0.9330 | 1.4885 |
| Total cholesterol | 1.1646 | 0.9016 | 1.5045 |
| Current smoking | 1.0476 | 0.6032 | 1.8194 |
| Diabetes mellitus | 2.9178 | 1.6814 | 5.0633 |
| >1 location of vascular disease | 3.1760 | 1.7611 | 5.7276 |
| Valve calcium present | 0.8717 | 0.5001 | 1.5193 |
| Shrinkage factor 0.7945. For continuous predictors, hazard ratios per 1SD are given | | | |

| **D** Model V (all calcium presence/absence combined) | | |  |
| --- | --- | --- | --- |
|  | **HR** | **95% CI lower limit** | **95% CI upper limit** |
| Age | 0.9662 | 0.6944 | 1.3444 |
| Age^2 | 0.9001 | 0.6901 | 1.1739 |
| Creatinine | 2.0031 | 0.8896 | 4.5105 |
| Sex | 1.1802 | 0.9338 | 1.4917 |
| Systolic blood pressure | 1.0426 | 0.7813 | 1.3912 |
| Total cholesterol | 1.2466 | 0.9706 | 1.6012 |
| Current smoking | 0.9930 | 0.5701 | 1.7296 |
| Diabetes mellitus | 2.7777 | 1.5762 | 4.8949 |
| >1 location of vascular disease | 2.7795 | 1.5346 | 5.0342 |
| Valve calcium present | 0.8463 | 0.4863 | 1.4727 |
| Thoracic aorta calcium present | 1.0424 | 0.5753 | 1.8888 |
| Coronary artery calcium present | 3.3719 | 1.2691 | 8.9590 |
| Shrinkage factor 0.7644. For continuous predictors, hazard ratios per 1SD are given | | | |

# Supplemental figure S1. Calibration plots of models with calcium presence/absence for the prediction of MACE+


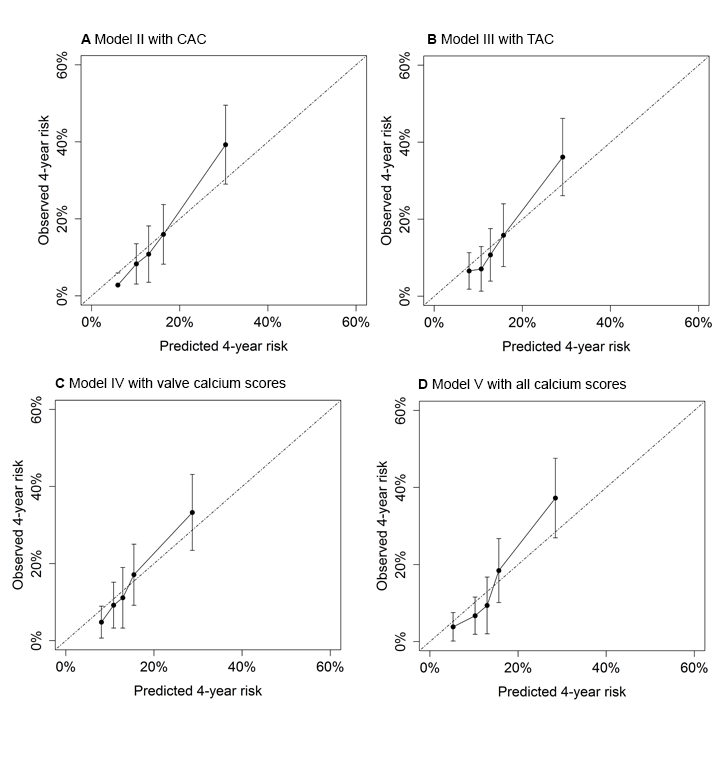


# Supplemental table S6. C-statistics and net reclassification index for models with and without calcium presence/absence as predictor for MACE+

|  | **C-statistics** | **Net reclassification index (NRI) (%)** | |
| --- | --- | --- | --- |
|  | **(95%CI)** | **Risk difference based NRI* (95% CI)** | **Categorical NRI****  **(95% CI)** |
| **Model 1**  No scores | 0.65  (0.59-0.72) | ref | ref |
| **Model II**  CAC score | 0.68  (0.61-0.74) | **24.04**  **(8.26-38.83)** | **17.30**  **(1.98-32.31)** |
| **Model III**  TAC score | 0.65  (0.59-0.71) | -10.25  (-22.21- 0.82) | 0.79  (-5.73-6.65) |
| **Model IV**  Valve scores | 0.65  (0.58-0.71) | **-14.58**  **(-27.11- -3.17)** | 0.32  (-9.64-11.23) |
| **Model V**  All scores | 0.67  (0.61-0.72) | 8.13  (-12.02-29.24) | 12.54  (-2.32-28.53) |
| * A cut-off value of 2% was used for the risk difference based reclassification index  ** Categories for the categorical were based on 10-year risk categories <20%, 20-30%, 30-40%, and >40% translated to 4-year risks: <9%, 9-13%, 13-18%, >18%. | | | |

**References**

1. Agatston AS, Janowitz WR, Hildner FJ, Zusmer NR, Viamonte M, Jr., Detrano R. Quantification of coronary artery calcium using ultrafast computed tomography. J Am Coll Cardiol. 1990;15(4):827-32.

2. Budoff MJ, Mao S, Takasu J, Shavelle DM, Zhao XQ, O'Brien KD. Reproducibility of electron-beam CT measures of aortic valve calcification. Acad Radiol. 2002;9(10):1122-7.

3. Austin PC, Lee DS, Fine JP. Introduction to the Analysis of Survival Data in the Presence of Competing Risks. Circulation. 2016;133(6):601-9.

4. Steyerberg EW. Clinical prediction models: a practical approach to development, validation and updating. New York, USA: Springer. 2009.

5. Cook NR, Ridker PM. Advances in measuring the effect of individual predictors of cardiovascular risk: the role of reclassification measures. Ann Intern Med. 2009;150(11):795-802.

6. Steyerberg EW, Pencina MJ. Reclassification calculations for persons with incomplete follow-up. Ann Intern Med. 2010;152(3):195-6; author reply 6-7.

7. Pencina MJ, D'Agostino RB, Sr., Steyerberg EW. Extensions of net reclassification improvement calculations to measure usefulness of new biomarkers. Stat Med. 2011;30(1):11-21.

8. Steyerberg EW, Vickers AJ, Cook NR, Gerds T, Gonen M, Obuchowski N, et al. Assessing the performance of prediction models: a framework for traditional and novel measures. Epidemiology. 2010;21(1):128-38.

9. Simons PC, Algra A, van de Laak MF, Grobbee DE, van der Graaf Y. Second manifestations of ARTerial disease (SMART) study: rationale and design. Eur J Epidemiol. 1999;15(9):773-81.

10. Kaasenbrood L, Bhatt DL, Dorresteijn JAN, Wilson PWF, D'Agostino RB, Sr., Massaro JM, et al. Estimated Life Expectancy Without Recurrent Cardiovascular Events in Patients With Vascular Disease: The SMART-REACH Model. J Am Heart Assoc. 2018;7(16):e009217.

11. Anand SS, Caron F, Eikelboom JW, Bosch J, Dyal L, Aboyans V, et al. Major Adverse Limb Events and Mortality in Patients With Peripheral Artery Disease: The COMPASS Trial. J Am Coll Cardiol. 2018;71(20):2306-15.

12. Dorresteijn JA, Visseren FL, Wassink AM, Gondrie MJ, Steyerberg EW, Ridker PM, et al. Development and validation of a prediction rule for recurrent vascular events based on a cohort study of patients with arterial disease: the SMART risk score. Heart. 2013;99(12):866-72.
